# Twist again: Dynamically and reversibly controllable chirality in liquid crystalline elastomer microposts

**DOI:** 10.1126/sciadv.aay5349

**Published:** 2020-03-27

**Authors:** James T. Waters, Shucong Li, Yuxing Yao, Michael M. Lerch, Michael Aizenberg, Joanna Aizenberg, Anna C. Balazs

**Affiliations:** 1 Chemical Engineering Department, University of Pittsburgh, Pittsburgh, PA 15261, USA.; 2 Department of Chemistry and Chemical Biology, Harvard University, Cambridge, MA 02138, USA.; 3 Wyss Institute for Biologically Inspired Engineering, Harvard University, Cambridge, MA 02138, USA.; 4 John A. Paulson School of Engineering and Applied Sciences, Harvard University, Cambridge, MA 02138, USA.

## Abstract

Photoresponsive liquid crystalline elastomers (LCEs) constitute ideal actuators for soft robots because their light-induced macroscopic shape changes can be harnessed to perform specific articulated motions. Conventional LCEs, however, do not typically exhibit complex modes of bending and twisting necessary to perform sophisticated maneuvers. Here, we model LCE microposts encompassing side-chain mesogens oriented along a magnetically programmed nematic director, and azobenzene cross-linkers, which determine the deformations of illuminated posts. On altering the nematic director orientation from vertical to horizontal, the post’s bending respectively changes from light-seeking to light-avoiding. Moreover, both modeling and subsequent experiments show that with the director tilted at 45°, the initially achiral post reversibly twists into a right- or left-handed chiral structure, controlled by the angle of incident light. We exploit this photoinduced chirality to design “chimera” posts (encompassing two regions with distinct director orientations) that exhibit simultaneous bending and twisting, mimicking motions exhibited by the human musculoskeletal system.

## Teaser

Simulations show that a liquid crystalline elastomer post can be made to twist with both left and right chirality with light.

## INTRODUCTION

The human musculoskeletal system can undergo a remarkable range of motion, including movements that combine bending and twisting. Our daily activities, as well as many yoga poses and dance moves, would not be possible without this degree of flexibility. While there have been tremendous advances in soft robotics (*1*, *2*), it remains a substantial challenge to design materials that can actuate the simultaneous flexing and turning needed to perform complex maneuvers. Currently, robots can only accomplish such coupled flexural and torsional behavior with the aid of pumps or motors (*3*–*5*). Materials that undergo a full range of dynamic motion in response to simple inputs from environmental cues are vital for developing responsive soft robots that operate without extensive tethers and wires (*6*–*8*). Here, we use computational modeling to design liquid crystalline elastomer (LCE) microscopic posts that permit new modes of actuation by exhibiting molecularly-encoded symmetry breaking. These elastomeric posts are inherently achiral but controllably form chiral structures of both handedness in the presence of light. In effect, the macroscopic chirality and particular handedness of the posts can be dynamically and repeatedly switched “on” and “off.” An experimental manifestation of the modeled system showcases the possibility for using light to induce symmetry breaking and achieve reversible clockwise and counterclockwise twisting of the same microposts. [It is noteworthy that while chiral dopants can be used to switch the handedness of cholesteric liquid crystals (*9*), the latter materials are themselves inherently chiral, as opposed to the initially achiral system considered here.] Few synthetic materials exhibit such bulk-scale induced and reversible chirality (*10*–*15*). We exploit this distinctive photoresponsive behavior to design the LCE posts that undergo combinations of bending and twisting necessary for executing complicated dynamic actions.

On a fundamental level, our study helps elucidate mechanisms that permit transduction across different energy domains within a single material. Namely, the bending and twisting of the LCEs involves a coupling among chemical, optical, and mechanical energy in the micropost. The absorption of ultraviolet (UV) light triggers the photoisomerization of azobenzene cross-linkers that, in turn, affects the nematic ordering and thereby alters the distribution of polymer chain conformations within the elastomer. The latter changes ultimately result in a macroscopic deformation of the post. Understanding how to design systems encompassing this level of integration is necessary for creating adaptive materials that produce beneficial responses to changes in the local environment. These materials systems can thus facilitate the fabrication of responsive robotic devices that exhibit controllable, dynamic behavior without the need for complex electronic components. In addition, the system does not necessitate the fabrication of a bilayer geometry (*16*–*18*), which is commonly used to preprogram morphological transformations, and requires the coordinated motion of two compatible materials. Rather, the fundamental properties of the LCE alone permit the material itself to be “programmed” to perform useful and versatile actions.

The elastomers modeled here are fabricated from monomers containing side-chain mesogens, which are aligned along a specific direction by an applied magnetic field (*19*). [Notably, compared to the additive manufacturing of LCEs in which the mesogens can only be oriented parallel to the printing direction (*20*), the use of a magnetic field before polymerization allows an arbitrary mesogen orientation prescribed to the LCE by the external field.] With the addition of an azobenzene cross-linker, the material forms a photoresponsive LCE, where the magnetically induced mesogen orientation is now “locked” into the matrix and specifies the nematic director of the sample. LCEs containing photoresponsive azobenzene molecules constitute ideal actuators for soft robots because the azo compounds undergo a *trans-cis* isomerization in the presence of light that disrupts the nematic ordering of the mesogens and thereby causes the material to undergo macroscopic shape changes (*21*–*23*). The shape changes, in turn, can be harnessed to perform mechanical work, as characterized by the strain in the material. The resultant conversion of light into work can be exceptionally high, with certain azo-LCEs displaying 100% increases in strain when the sample is illuminated (*21*). Light provides a particularly effective stimulus for regulating the work produced by microposts because the illumination can be applied remotely, readily turned “on” and “off”, focused on a specific area of the microstructure, or dynamically modulated around the sample (e.g., in a continuously rotating manner).

In previous studies, we developed a computational approach to simulate the response of surface-anchored LCE microplates to variations in temperature and observed quantitative agreement with the corresponding experiments. In the following studies, we augment our computational approach to model the post’s dynamic response to rays of penetrating light, which subsequently illuminates different portions of the microstructure as the post moves toward or away from the light source. Hence, we can capture the dynamic opto-chemo-mechanical interactions in the system. Because these dynamics depend, in complex ways, on the director orientation, the size of the anchored posts, and the incident angle of light, such calculations are essential for pinpointing the parameter space that provides the most beneficial, desired structural reconfiguration. Moreover, our approach allows us to design novel “chimera” or “dual-domain” structures, where the top half of the post (the “head”) encompasses one director orientation, and the anchored bottom half (the “body”) is inscribed with a different orientation of the director. As shown below, these chimera posts exhibit simultaneous flexural and torsional deformations that could allow “robotic materials” to ultimately undergo the range of motion exhibited by human musculoskeletal system.

## RESULTS

### Theoretical modeling

**Fig. 1:**
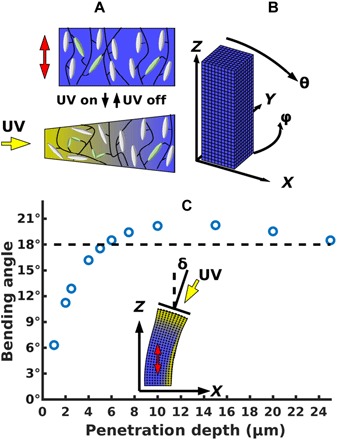
Schematic of the system and calibration of light penetration parameter. (**A**) Schematic of the polymer network. The micropost is composed of a network of polymer chains. Mesogens (gray) are attached side-on to the polymer backbone (black), and the chains are cross-linked by an azobenzene derivative (green). The orientation of the nematic director is indicated by the double-headed red arrow. Under UV light, the cross-linkers undergo a *trans-cis* isomerization, disrupting the local nematic order. This effect attenuates deeper into the material. (**B**) Schematic of the post. The elastomeric micropost is modeled with a set of initially cubic finite elements, at a scale of one element per 2.5 μm. The incident light source can be varied in both the polar (θ) and azimuthal directions (ϕ). (**C**) Calibration of light-scattering distance. The penetration of light was assumed to fall off exponentially, with a length scale chosen to match experimental results of an 18° deflection for light incident at 30° from vertical, for a micropost of height 75 μm and width 25 μm. From this comparison, we obtain a value of 6 μm for the penetration depth. The inset shows the bending angle (δ) measured by the deflection of the surface normal at the top of the post relative to its initial vertical orientation.

Within the LCEs, the mesogens are attached side-on to the polymer backbone, as shown in Fig. 1A. The alignment of the liquid crystal mesogens produces a corresponding anisotropy in the conformations of the polymer chains. By cross-linking the chains while the mesogens are in the aligned, nematic liquid crystal state, the resulting elastomer exhibits the specific nematic director orientation present at the time of cross-linking (*19*). Disrupting the nematic ordering through heating (*23*, *24*) or UV light induces a contraction along this director and an expansion in the two orthogonal directions. The original state of the system, however, is recovered upon removal of the stimulus.

Our continuum model of LCEs allows us to capture the macroscopic response of the material due to conformational changes of the constituent polymer chains (*19*). Within this framework, we use the Gent model (*25*) for rubber elasticity to describe the resistance of the material to shear deformations. This model accounts for the finite extensibility of the polymer chains (as well as the thermally induced entropic contraction of rubbery materials) and thereby characterizes the behavior of responsive elastomers more accurately than the simpler neo-Hookean model (*26*). The resistance of these materials to compression is added to the Gent model through an empirically derived term (*27*, *28*); consequently, the elastic properties of the elastomers are described by the following free energy

$$F=\mu \text{log}(\frac{J_{m}-I_{1}}{J_{m}-3})-\mu \text{log}J+\frac{\kappa }{2}{\text{log}}^{2}J$$ (1)

where μ represents the shear modulus of the material and κ represents the bulk modulus. *I*_1_ is the first invariant of the strain tensor, and *J* is the square root of the third invariant, corresponding to changes in volume. The parameter *J_m_* represents the limit of the extensibility of the polymer chain, typically chosen to be 30 times the relaxed extension. Together, the first two terms describe the resistance of the material to shear deformations, while the third term represents the resistance of the material to volumetric changes.

An additional term, *F*_nem_, is needed to describe the coupling of the nematic order to the strain tensor. The degree of nematic ordering is characterized by the scalar order parameter *S*. A change in the nematic ordering, Δ*S*, alters the conformational biasing of the polymer chains in the elastomer and produces a change in the shear deformation. The simplest form of *F*_nem_ is given as (*29*)

$$F_{\text{nem}}=\frac{\alpha }{2}\Delta Q_{\mathrm{ij}}\varepsilon_{\mathrm{ij}}$$ (2)

Here, ε*_ij_* is the left Cauchy-Green strain tensor, corresponding to the material frame coordinates. The change in the nematic tensor Δ*Q* is expressed as

$$\Delta Q_{\mathrm{ij}}=\Delta S(n_{i}n_{j}-\frac{1}{3}\delta_{\mathrm{ij}})$$ (3)

where *n_i_* and *n_j_* are the components of the nematic director $\hat{n}$ and δ is the Kronecker delta. The nematic director is fixed in the material frame, so the director rotates with the material as the sample is deformed by external stimuli. The constant α is a measure of the coupling between the nematic order and the material deformation. The value of αΔ*S* can be obtained through a fit to experimental measurements of the relative contraction and expansion of the LCE upon heating to the isotropic state.

In the computational model, the unit vector $\hat{n}$ is assumed to be constant over the course of the simulations; only the scalar order parameter Δ*S* undergoes changes, reflecting the difference in the nematic ordering of the sample relative to that in the “as-prepared” material. Hence, the resulting shape deformations of the LCE micropost reveal the director orientation initially programmed into the LCE. Because the material was in the liquid crystalline phase when it was cross-linked, the value of Δ*S* becomes negative when the material enters an isotropic state.

As noted above, the nematic director is locked into the elastomer by the applied magnetic field; this justifies our assumption that the director remains fixed in the frame of the material. In other words, a nematic director that points along the axis linking two mesh nodes will remain oriented along the axis between these nodes, even as the coordinates of those nodes in the laboratory frame (where bending and twisting are measured) might shift or rotate. [If the sample were to be cross-linked in the isotropic phase, then the director reorientation would play a more important role in the material’s behavior (*30*).]

Here, we augment our previous computational model for the behavior of LCEs to include the effects of an azobenzene derivative in the prepolymer mixture. In response to UV light, this photoresponsive cross-linker undergoes a *trans-cis* isomerization, which disrupts the nematic order in the illuminated region. This isomerization, however, is localized near the illuminated surface because light only penetrates the material to a finite depth. Consequently, the effect of the light on the change in the order parameter can be written as (*31*)

ΔS(r)=−exp(−l/λ) (4)

The variable *l* is the path length of light through the material up to the point where the order parameter is evaluated. The light penetration is assumed to decay exponentially over a length scale λ, which can be empirically determined by fitting the bending response of our simulated LCE posts to experimental measurements. Namely, by matching the experimentally observed degree of bending with values obtained from comparable simulations, we obtain a value for the unknown depth parameter λ in Eq. 4. We also calculate this parameter by using the Beer-Lambert law; this calculation yields an attenuation length of approximately 4 μm (see the Supplementary Materials).

The path length of light, *l*, is computed through a ray-tracing algorithm; this path length is found for each point in space where the free energy is calculated (i.e., the “query points”). The value of *l* is obtained by iterating over a list of mesh node triplets that define external faces of the simulated post and identifying any intersections between the external surface of the post and the rays extending from the query point toward the light source. All the rays from the light source to the material are assumed to be parallel, as the distance from the micropost to the light is several orders of magnitude larger than the size of the post in the experimental setup. In addition, in this study, we neglect the variance of the index of refraction within the material.

When the light source is removed, the polymers relax, and the previously illuminated region of the elastomer returns to its ordered state. While the photoresponse of an individual azobenzene molecule can occur on a time scale of 10^−9^ s or less (*32*), the resulting mechanical equilibration of the microposts occurs on a time scale of minutes (*19*), longer than can be simulated in a reasonable time frame, so we omit the specific details of the excitation and relaxation rates. Diffusion of heat across the dimensions of the micropost is expected to occur on a time scale of several microseconds to milliseconds (*33*), allowing us to ignore thermal gradients within the material. By treating these processes as instantaneous, we obtain the final conformations of the illuminated posts in computationally reasonable time scales.

### Light-induced deformations of LCE microposts

Experiments to probe the response of LCE microposts to UV light were performed on a specific set of cases (*19*), where posts of a particular size encompassed a nematic director oriented perpendicular to the substrate. These studies, however, did not consider how modifying the height and width of the post or altering the director orientation in the material affects the sample’s response to light. Below, we first use the known experimental data to calibrate our simulations and, through these simulations, establish how varying salient features of the posts affects the system’s behavior, allowing us to pinpoint conditions where nonchiral posts can be dynamically transformed into three-dimensional chiral structures. By simulating the behavior of dual-domain (“chimera”) posts, where each half of the sample incorporates a distinct director orientation, we also predict how a single material can undergo simultaneous bending and twisting, thereby extending the range of dynamic reconfigurability evidenced in synthetic materials.

#### Vertical orientation of the nematic director

Recent experiments (*19*) revealed that LCE microplates and microposts encompassing a vertically oriented nematic director will bend toward a light source under a certain intensity of irradiance for various incident angles of light. This experimental setup (*19*) involved posts 75 μm in height and 25 μm in width that were irradiated with nonpolarized light of intensity 3.5 mW/cm^2^. This intensity is sufficiently low that the response is dominated by photochemical effects, as opposed to photothermal (*34*), justifying the omission of heat conduction in our model. Figure 1B provides a schematic of the finite element representation of such a post; we vary the incident angle of light about the two axes indicated in the figure. We use this geometry to calibrate the parameters of our simulation with physical values from the experiments. Namely, we simulated the post’s response to a light source fixed at an angle 30° from vertical, for different values of the light penetration depth, λ. In agreement with the experimental findings, the simulations show that these posts bend toward the light. The light disrupts the organization of the mesogens along the illuminated face, as depicted in the schematic in Fig. 1A, where yellow indicates the most illuminated areas and blue marks the least exposed regions. The local disruption of mesogen alignment causes the exposed face to shrink vertically; in the nonexposed regions, however, the original height of the post is upheld by the persistence of the nematic order and extension of the mesogens along the perpendicular direction. The inset in Fig. 1C shows that the contraction of the illuminated face tilts the top surface toward the light. Notably, the post does not twist out of the central plane that cuts through the length of the post.

The degree to which the post bends depends on the incident angle of the light and the penetration depth λ. The points on the plot in Fig. 1C indicate the simulation results for the bending of the posts (in the *xz* plane) at different values of λ when the light source is fixed at 30° from vertical. The simulated posts consisted of a 3751-node mesh, defining the vertices of 3000 finite cubic elements, which initially were 2.5 μm on each edge. This mesh size was chosen after simulating the post deflection at a range of resolutions (representing initially cubic volumes between 9 and 1 μm on a side) and noting the convergence of the data for values of the bending angle (see the Supplementary Materials). The simulations reveal that at small values of λ, increasing the penetration depth leads to an increase in the bending of the posts toward the light. With the angle of incident light fixed at 30°, the experimentally observed (*19*) deflection of the posts was equal to 18°, as marked by the dashed red line. The intersection of the curve for the simulated data and the dashed line occur at 6 μm. In other words, the best fit between the simulations and experiment occurs at λ = 6 μm. Repeating this simulation at a finer resolution revealed no significant change in the results. Notably, this value is comparable to the theoretical expectation of 4 μm obtained from Beer-Lambert law (see the Supplementary Materials). As the penetration depth is increased beyond this λ value, the bending response eventually decreases because the affected region becomes sufficiently broad that it encompasses the entire post and thus the post no longer exhibits the preferential contraction that leads to the tilting, instead exhibiting a vertical shrinkage and outward expansion.

**Fig. 2:**
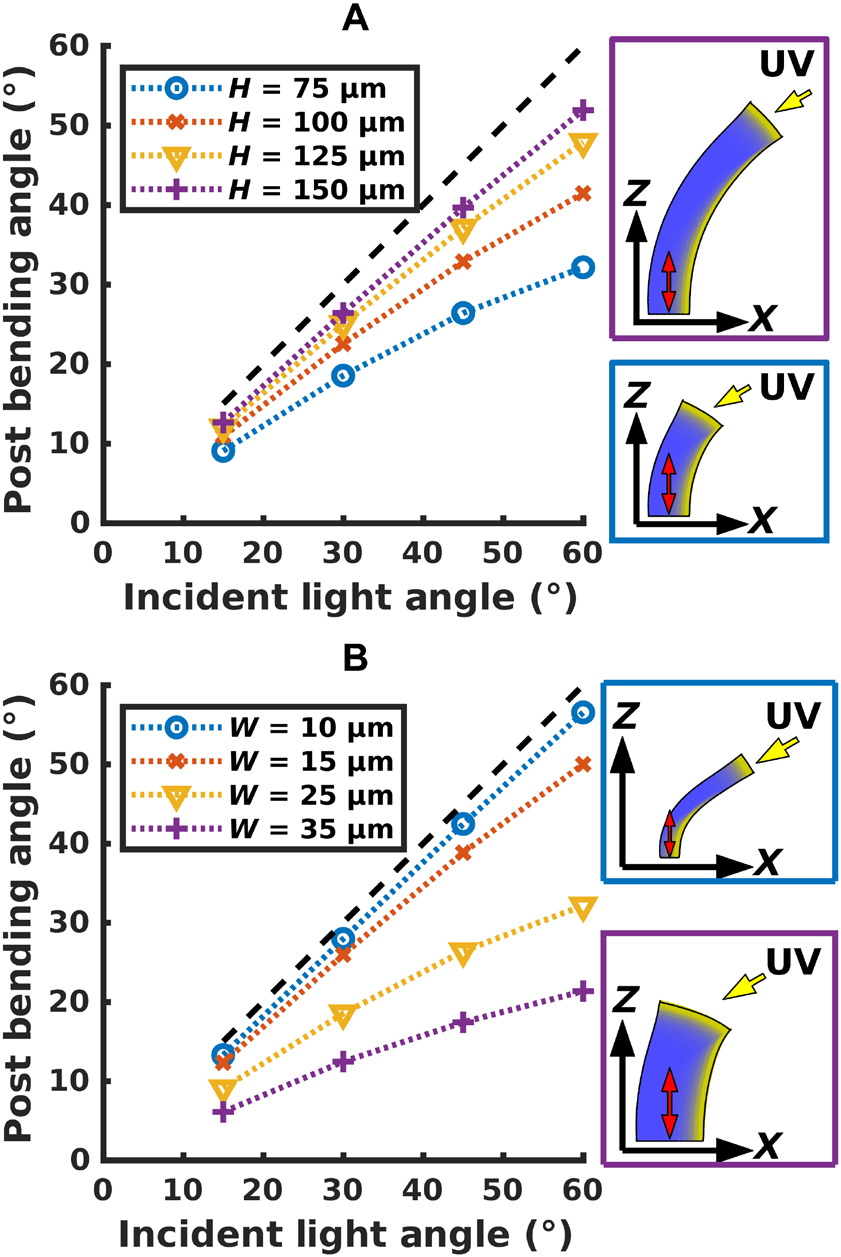
Effect of post aspect ratio for director orthogonal to substrate. (**A**) Effect of micropost height on bending response. The height ranges from 75 to 150 μm, while width is kept at 25 μm. Increasing the height over which the post can bend leads to a larger bending response. This is bounded above by the angle of the incident light source from vertical, indicated by the dashed line. (**B**) Effect of micropost width on bending response. At a fixed height of 75 μm, we vary the post width between 10 and 35 μm. Thinner posts have a lower bending modulus and are thus able to track the light more closely, although the self-shadowing of the post prevents it from bending past the incident angle of the light.

Using this calibrated model, we computed the effect of varying the geometry of the microposts on the bending response of this microstructure. In particular, we increased the aspect ratio (height divided by width) and found that increasing the post height increases the post’s degree of bending across a range of incident light angles (Fig. 2A). As the height and width of the posts were varied, we maintained a consistent ratio between the number of finite elements and the simulated volume. However, this behavior is bounded by the self-shadowing of the post; once the post has tilted such that the top surface is orthogonal to the incident light, the post lies outside the illuminated area and, hence, will not continue bending. Thus, the deflection of the top surface of the post never exceeds the angle of the incident light, measured from the vertical axis, as marked by the dashed lines in Fig. 2 (A and B). Experimentally, the height of the post is practically limited to 150 μm by the fabrication process; therefore, we limited the maximum height of the posts in the simulations to this value.

Decreasing the thickness of the posts decreases the bending modulus and thus also increases the bending response (Fig. 2B). In addition, the region affected by the UV light represents a greater fraction of the total cross section of a thin post. This trend will be limited, as eventually the post thickness approaches the penetration depth of the UV light into the elastomer, and the bending response will decrease as the disruption of the nematic phase occurs throughout the post rather than just being localized to one side. In the fabricated system, there will be an additional lower bound imposed by the alignment of mesogens along the wall for chains that are sufficiently close to that bounding layer. This anchoring effect is not presently included in our computational model. Namely, below a certain thickness, the influence of the surface anchoring of the mesogens to the walls of the mold will dominate over the effect due to the applied magnetic field. We expect the lower bound from this anchoring effect to be greater than that imposed by the penetration effect. By restricting our further studies to posts of width at least 25 μm, we can avoid this regime where experimental and simulation results are expected to diverge.

#### Orientation of the nematic director parallel to the substrate

**Fig. 3:**
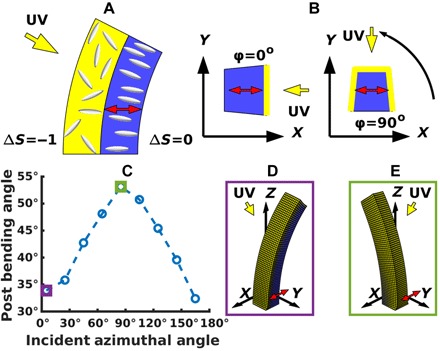
Light-phobic bending for director in the plane of the substrate. (**A**) Schematic of light-phobic bending. Disruption of the nematic state near the illuminated face will cause that region to expand vertically when the director is in the plane of the substrate. This will, in turn, cause the post to bend backward, away from the light. (**B**) Rotational variation of response. An overhead view of the post shows that we now have an additional degree of freedom as the light source is rotated relative to the director. To test the dependence on this coordinate, we simulate multiple configurations where the nematic director (red arrows) is oriented along the *x* axis and the light source (yellow arrows) is rotated around it. (**C**) Dependence of bending on incident azimuthal angle. Simulations represent posts of height 150 μm and width 25 μm, responding to a light source 30° from vertical. The light-averse response is strongest when the light is orthogonal to the programmed nematic director. (**D**) Post at incident azimuthal angle of 5°. Here, the light is incident at an oblique angle to the nematic director and induces a small backward bending. (**E**) Post at incident azimuthal angle of 85°. Here, the light is nearly orthogonal to the nematic director and induces a larger degree of backward bending than for an azimuthal angle of 5°.

Microplates with a rectangular cross section (being relatively narrow along the *x* direction and wide along the *y* direction) have been fabricated (*19*); however, the experimental studies did not extend to microposts (with square cross section) having a nematic director parallel to the substrate. Simulations of the latter system reveal that the posts display a unique light-averse behavior (Fig. 3) compared to the toward-the-light bending exhibited by the posts with the vertical director orientation. These simulations were run with cubic elements initially 5 μm on a side, with the entire post (150 μm × 25 μm × 25 μm) comprising 750 elements and 1116 nodes. This lower resolution was necessary to obtain convergence of the results in a computationally reasonable time frame. In contrast to the scenarios described above, if the programmed director lies parallel to the substrate, then disruption of the nematic order in the illuminated face causes the post to expand in the vertical direction and bend backward, away from the light (shown schematically in Fig. 3A). Notably, the bending of the post is not limited by self-shadowing, allowing the posts with high aspect ratios to bend beyond 90°.

Orienting the director away from the symmetry axis in the plane of the substrate breaks the rotational symmetry present when the director is aligned with the vertical axis of the post. Hence, the posts should display a variation in their response to light as the direction of the incident beam is rotated about this structure (Fig. 3B) at a constant angle of 30° from vertical. This variation can be seen in Fig. 3C; for a nematic director oriented along the *x* axis, the post displays greater bending away from the light source for light directed in the *yz* plane (90°) versus the *xz* plane (0°, 180°). Namely, at 90°, the angle of incident light lies orthogonal to the director; moreover, the illumination falls on three sides of the structure (see right image in Fig. 3B). Both these factors cause larger perturbations in the mesogen ordering and, hence, contraction of the material. On the other hand, when the light lies in the *xz* plane, some component of the light lies along the director (see left image in Fig. 3B) and less of the sample is perturbed by the light. The bending angles found from simulations at 5° (purple square) and 85° (green square) are indicated on the graph, and the corresponding final post conformations are displayed in Fig. 3 (D and E, respectively).

The effect of increasing the post aspect ratio is qualitatively similar to that found for the cases encompassing the vertical director. In particular, increases to the post height and decreases in post width both produce a larger bending response for the microposts. While the simulation results predict that this trend continues as posts become thinner, LCE microplates in the experiments demonstrate decreased response below a width of 35 μm. The anchoring of the nematic mesogens at the surface of the plates prevents them from following the orientation prescribed by the magnetic field. This anchoring effect will be less noticeable for the planar director orientation or the skewed orientation discussed below versus the vertical orientation where the director generally lies parallel to the faces of the post. Note that in all the cases discussed above, the posts did not twist out of the central *xz* plane.

#### Angled orientation of the nematic director

**Fig. 4:**
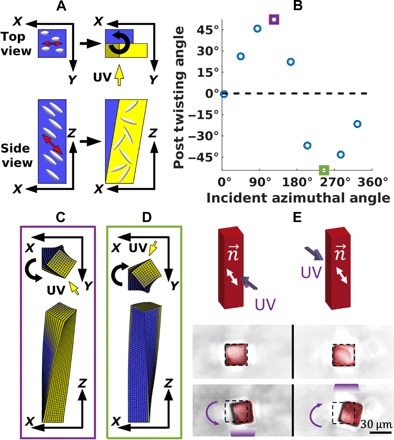
Twisting behavior for director at 45° from the substrate. (**A**) Schematic of illuminated post. When the illuminated region transitions from a nematic to an isotropic state, the angled orientation of the director will cause it to deform into a parallelogram. This will produce a shearing at the top of the post, causing it to twist. For this particular angle of the incident light, the induced twist is right-handed, as indicated by the curved arrow in the top view. (**B**) Micropost twist angle versus azimuthal angle of incident light. As the light source is moved counterclockwise around the post, it first produces right-handed (positive) twisting and then left-handed (negative) twisting, with no twisting at 0° or 180° from the *x* axis. (**C**) Overhead and side view of the micropost for an incident azimuthal angle of 125°. Here, light incident on the +*y* face produces right-handed twisting. This corresponds to the data point outlined in purple in (B). (**D**) Overhead and side view of micropost for an incident azimuthal angle of 245°. Here, light incident on the −*y* face produces left-handed twisting. This corresponds to the data point outlined in green in (B). (**E**) Experimental observations of twisting of surface-anchored LCE microposts. For the director orientation of 45° from the vertical, the LCE microposts reversibly twist clockwise and counterclockwise, with handedness controlled by the direction of incident light, as predicted by the simulations.

Whereas director orientations along the vertical axis of the post or in the plane of the substrate can produce a vertical contraction or expansion of the illuminated face, respectively, a director orientation rotated 45° from vertical leads to the structural change illustrated in Fig. 4A and quantified in Fig. 4B. In particular, when the local nematic order is disrupted, the illuminated region becomes tilted, shifting from a rectangle to a parallelogram (see side view in Fig. 4A). This particular deformation causes a shearing between the illuminated and nonilluminated sides of the post and thus produces a twisting of the top surface relative to the fixed base. This morphology is evident from the simulation results shown in Fig. 4 (C and D). The direction of this twisting depends on which of the faces of the micropost is exposed to the UV light source. As shown below, both right- and left-handed twisting can be produced by stimulating opposite faces of the post.

We simulated an LCE micropost with height 150 μm and width 25 μm exposed to a light source incident at 86° from vertical using a mesh of 6000 elements and 7381 nodes. This mesh size was chosen to maintain the same resolution of the post volume as in the case of the 75-μm posts represented with 3000 elements. The nematic director was uniform throughout the post, oriented along the vector $(\hat{x}+\hat{z})/\sqrt{2}$, and the azimuthal angle of the incident light about the post was varied in 40° increments starting at 5° from the *xz* plane up to 325°. Figure 4B shows that the maximum twist occurs beyond 90° at the 125° data point, outlined with a purple square. The displacement of this maximum from 90° is due to the fact that as the post twists, it exposes a different face to the incident light. While directing the incident light along the *y* axis (90°) maximizes the twisting near the fixed base of the post, the rate of twisting decreases away from the substrate where the post is already twisted (see the Supplementary Materials). Therefore, it is more effective to rotate the light source beyond 90° in the direction of the twist. As twisting is induced in the post, the director (fixed in the material frame) is gradually rotated so it is nearly orthogonal to the incident light along the height of the post. The resulting conformation of the micropost is shown in Fig. 4C. We find that an incident angle of 125° from the *x* axis maximizes the total twist over the height of the post for the given dimensions.

For the maximum twist (125° azimuthal angle and 86° polar angle), we estimate that the post experiences a torque of 2.3 × 10^−10^ N m. This estimate is obtained from an expression relating the torque to the material’s twist and its resistance to deformation.

$$\tau =\mu J_{p}\frac{\varphi }{L}$$ (5)

Here, μ is the shear modulus (570 kPa), *J_p_* is the cross-sectional moment, equal to 1/6 *W*^4^ for a square post (where the width *W* is 25 μm), and φ/*L* is 1.14 rad/150 μm. Thicker posts lead to a lower twist but a larger torque due to the *W*^4^ dependence. For instance, a post of width 35 μm twists 0.75 rad, which is equivalent to 7.1 × 10^−10^ N m, approximately three times larger than the value for the 25-μm width post. Some of this increase is due to the fact that the thicker post has 40% more surface area that can be impinged by light. In addition, the moment arm for the torque is larger, as the force twisting the post is applied 40% further from the center for the wider post versus the thinner post.

This twist can take on both right-handed (positive) and left-handed (negative) values. The chirality in this system is not intrinsic to the material but is determined by the azimuthal angle of the incident light relative to the director. By altering the angle of the light, we can change the handedness of the twisting. The crossover between the different directions of rotation occurs when the incident light and the nematic director are coplanar, at 0° and 180°, as seen in Fig. 4B. A left-handed twist geometry is shown in Fig. 4D, corresponding to the data point at 245° indicated by the green square in Fig. 4B.

By fabricating an array of LCE microposts, we found that irradiation of the posts with UV light at different angles of incidence triggers a twisting behavior that closely follows the predictions from the simulations. In particular, Fig. 4E shows that illumination aimed at the front side of the micropost causes a right-handed twisting, while illumination from the opposite direction causes a left-handed twisting of the same microstructures. As predicted, the twisting is accompanied by a small bending of the posts in the plane to the director. In the experimental system, however, the observed degree of twisting about the vertical axis (~22°) is smaller than the calculated values (~54°). This discrepancy can be due to the fact that the simulations do not describe the misalignment of the mesogens along the outer surfaces of the microposts, as mentioned above in relation to Fig. 2.

**Fig. 5:**
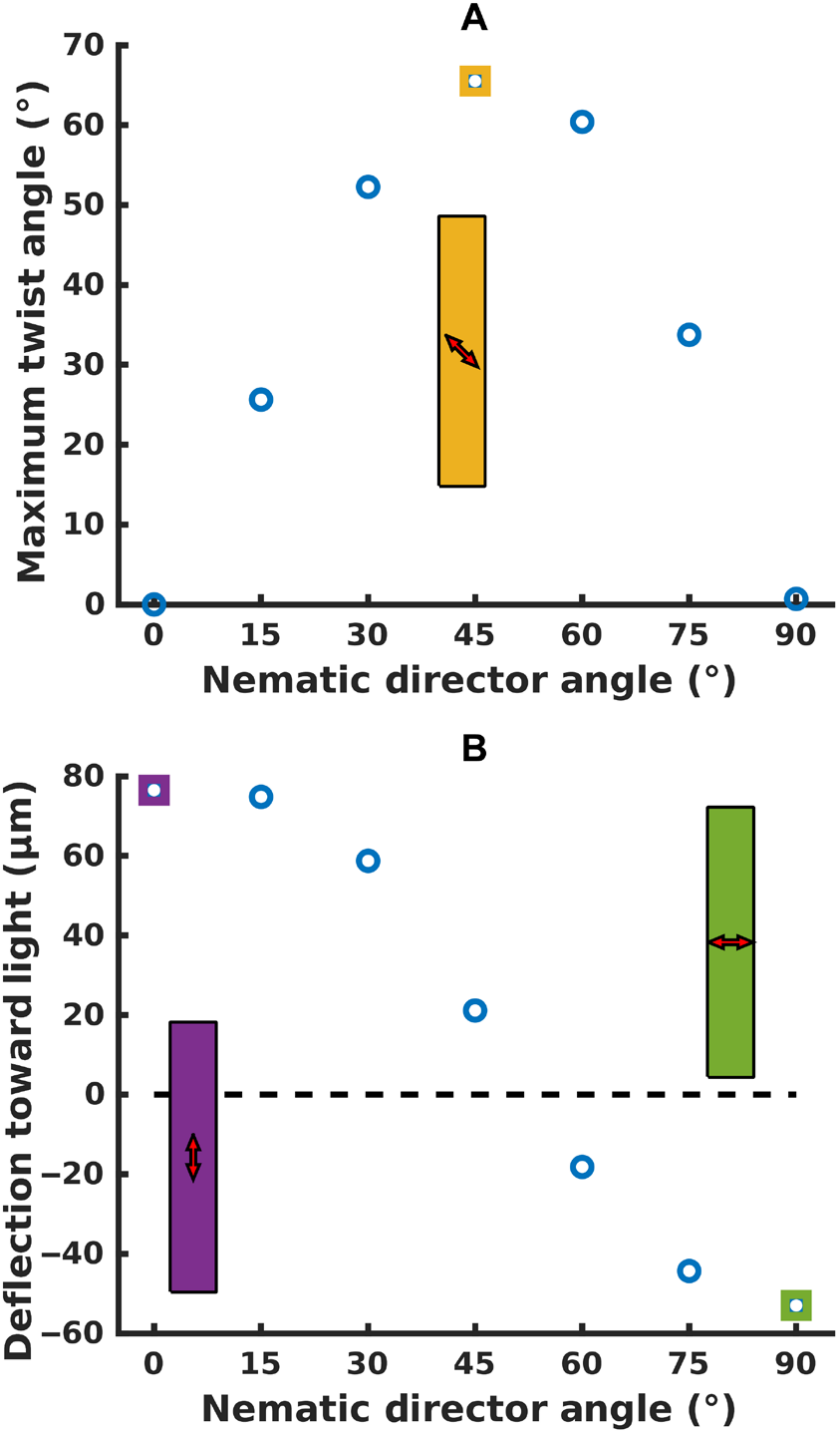
Effect of director tilt angle on twisting and bending. (**A**) Twist angle versus nematic orientation. As the director orientation is varied between 0° (vertical) and 90° (planar), we see that the twisting behavior of the micropost measured at an azimuthal angle of 125° from the *x* axis first increases and then decreases. The maximum twist is achieved for a nematic director near 45° from the vertical axis. (**B**) Deflection of posts toward and away from light. The bending response of the micropost moves from light-seeking to light-avoiding as we consider nematic director orientations ranging from vertical (purple) to planar (green). The cross-over point, between 45° and 60° from vertical, coincides with the maximum twisting orientation.

Having demonstrated controllable twisting behavior in the LCE microposts, we determine the nematic director orientation that maximizes the extent of this twisting response. We simulated an LCE micropost with different orientations of the skew angle of the nematic director, ranging between vertical and planar, while sweeping the azimuthal angle of the incident light around the post. Figure 5A depicts a cross section of these results where the twisting behavior is most prominent (at a fixed azimuthal angle of 125° from the *x* axis and polar angle of 86° from the *z* axis). The greatest amplitude of twist occurs when the nematic director is oriented between 45° and 60° from vertical.

**Fig. 6:**
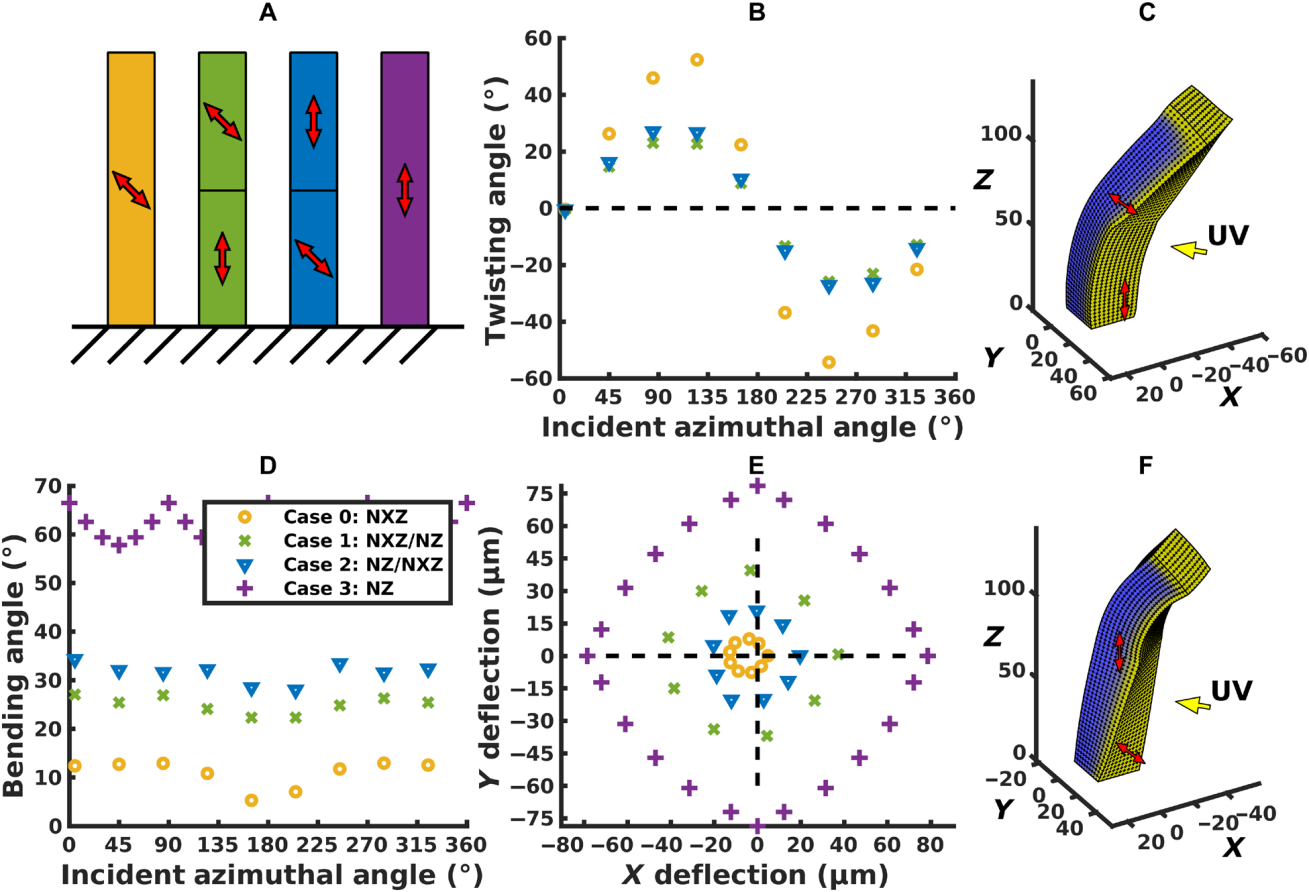
Twisting and bending behavior in chimeric microposts. (**A**) Schematic of chimeric microposts. Bi-domain microposts are constructed with a vertical director at the base and a tilted director in the top half (green), as well as the reverse geometry, with the tilted director at the base (blue). Tilted (yellow) and vertical (purple) monodomain microposts are presented for comparison. (**B**) Micropost twisting angle versus incident azimuthal angle. The light source is at a polar angle of 86° from vertical. A tilted nematic in the bottom half (blue points) reduces the total twisting by 50% relative to the homogeneous tilted nematic (yellow points), as only half of the post is twisting. Placing the tilted nematic in the top half (green points) further reduces twisting as the bending at the base moves the top half out of the path of incident light. (**C**) Post with vertical director in anchored bottom half. The incident azimuthal angle is 125°. The post bends toward the light at the base and twists in the top half. (**D**) Bending angle as a function of azimuthal angle of incident light. The deflection angle at the top of the post is larger when the vertical director is in the free top half versus when it is in the constrained bottom half. (**E**) Deflection in the *xy* plane, measured at the top of the post. Bending at the base (green points) leads to a greater deflection than bending that begins halfway up the post (blue points). Both cases result in greater deflection than for a homogeneously tilted director (yellow points) but less than that of a vertical director (purple points). (**F**) Post with tilted director in anchored bottom half. The incident azimuthal angle is 125°. The post twists at its base and bends toward the light in the top half.

Given that a vertically oriented director produces a bending response toward the light source and a nematic director in the plane of the substrate causes the post to bend away from the light source, we anticipate that there is value of the nematic director between 0° and 90° where the deflection of the post switches from light-seeking to light-avoiding. We characterize this deflection response by the displacement of the center of the top of the post in the *xy* plane from its initial position. Figure 5B reveals that this crossover occurs between 45° and 60° from the vertical case. This point where the amplitude of deflection toward or away from the light source is minimal coincides with the maximal twist response. Notably, at incident azimuthal angles near 0° and 180°, the posts display only bending behavior and no twisting (see yellow circles in Fig. 6). Hence, this single micropost can undergo a range of motions that depend on the coupling of the nematic director and the orientation of the light.

#### Posts encompassing two distinct director orientations

Having demonstrated the bending behavior for the vertically oriented director and twisting behavior for the tilted director, we combine these two modes of deformation into a single microstructure to increase the range of motion and functionality of the post. To this end, we construct a single post composed of distinct regions of different director orientations. In particular, we simulate the behavior of dual-domain “chimera” posts with (case 1) a tilted director in the anchored, bottom half, and a vertically oriented director in the free, upper half and (case 2) the reverse configuration, with the vertical director in the bottom half and the tilted director in the upper half. A schematic of these assemblies is shown in Fig. 6A. Such multidomain structures can potentially be achieved experimentally by cross-linking samples with different magnetically aligned nematic directors.

We first consider the degree of twisting displayed by cases 1, 2, and a single-domain post with an angled director (“case 0”), where all the posts have the same overall height. The tilted director is angled at 45° from the vertical axis in all the cases. In case 1, where the angled director is in the bottom half, the chimera post twists 50% less than the single-domain post (Fig. 6B). Here, the segment of the chimeric post with the tilted director simply has half of the height over which to twist. In case 2, where the titled director is in the upper half, the twist is reduced to 40% of the value for a homogeneous post. With the vertically oriented director in the bottom half (case 1), the bending at the base of the post moves the top half out of the path of the incident light. Consequently, the upper domain is at an unfavorable orientation for the light to induce twisting (Fig. 6C), and twisting occurs with a lower pitch than in the homogeneous case. Conversely, when the angled director is programmed into the lower, constrained half of the post (case 2), the twisting segment of the post remains at a favorable orientation relative to the incident light and the twisting occurs with the same pitch, albeit only over half of the height of the post.

With respect to bending, the behavior of the posts can be characterized in two different ways. In the first sense, the bending can be described in terms of the change in the normal vector at the top surface of the post; this measurement is especially useful for determining the optimal director arrangement for posts that serve as deformable micromirrors. Figure 6D indicates that placing the vertically oriented director in the top half (case 1) produces a greater response, bending, on average, 8° more than the post with the reverse chimera geometry (case 2). Namely, the bending of the post is optimized when the vertically oriented director is in the free half, which displays the greater light-seeking response.

Alternatively, the bending can be characterized by the deflection of the post into the *xy* plane. This measure is useful for designing posts that could simultaneously guide and sustain fluid flow in a microchamber, in analogy with effective wind blades (*35*) that are bent and subtly twisted to optimize air flow. Using this second metric, the post with the vertically oriented director on the bottom (case 1; Fig. 6C) produces a twofold larger response (Fig. 6E) than the reverse case (case 2; Fig. 6F). Here, the advantage occurs because bending the post by a given angle near the base will always produce a larger deflection than bending by the same angle closer to the top of the post. Figure 6 (C and F) also shows that these chimera structures combine bending and twisting in a single microstructure.

By using two different metrics for the bending of the chimera posts, we further demonstrate that the desired deformation response can be obtained by tailoring the configuration of the directors within the materials and thus tailoring the microstructures for various applications. While the respective single-domain post displays a higher degree of twisting or bending than the chimera posts, the dual-domain structure is necessary for achieving the distinctive combination of bending and twisting that can lead to new functionality.

## DISCUSSION

We modeled the behavior of LCE microposts encompassing nematic director orientations that were imposed by an external magnetic field in a post anchored by one end to a solid substrate. The LCE was cross-linked with an azobenzene linker, which undergoes a *trans-cis* isomerization in the presence of light, thus making the elastomer photoresponsive. In particular, the applied light locally disrupts the prescribed alignment of the side-chain mesogens, creating spatial heterogeneity in the sample. The local misalignment of the mesogens causes the illuminated regions to deform along a specific axis that is dictated by the inscribed director orientation. The deformation of the illuminated regions relative to the nonilluminated portions gives rise to the distinctive shape changes of the microposts.

This study revealed nontrivial modes of deformation and provided guidance for optimal feature sizes, illumination angles, and director orientations to elicit specific behaviors. In particular, the simulations showed that changing the orientation of the director from vertical to parallel drives the posts to shift from a light-seeking to light-avoiding behavior.

As prepared, the micropost is inherently nonchiral for any value of the nematic order parameter. However, our simulations suggest that if the director is oriented at 45° with respect to the substrate, then applying the light at specific angles drives the post to form a three-dimensional chiral structure, which can exhibit both clockwise and counterclockwise twisting. Thus, the chirality of the material can be dynamically and reversibly altered. Moreover, the extent and direction of twisting can be tailored by varying the incident angle of the light. The subsequent experiments confirmed that the chirality of the same LCE posts can be switched from right- to left-handed by simply changing the direction of light.

We exploited this behavior to design “chimera” posts that combine a bending and twisting behavior, which can be controlled by tailoring the director orientation in the head and body of the post. The tails of seahorses are square prisms (*36*), which allow them to exhibit distinctive bending and twisting motion in both the lateral and dorsal directions. The square geometry of the posts considered here can also allow greater structural reorientation than that provided by a cylindrical post because the illuminated faces and corners can provide distinct contributions to the photoresponsive behavior.

Microstructures with controllable bending, twisting, and chirality can be used to greatly expand the range of motion exhibited by microscopic robots and devices (*37*). This degree of structural reconfiguration is also valuable for creating shape-changing biomedical devices and microactuators (*16*). The techniques used to fabricate such posts can be adapted to produce thicker and longer posts. The chirality of mesoscopic posts (millimeters and centimeters in size) can be readily felt by touch, and hence, these posts could form a vital component in haptic devices (*38*), allowing enhanced and potentially expanded modes of interaction at human-machine interfaces. These simulation results thus provide useful guidelines for experimentally realizing such LCE systems. Experiments with microstructures fabricated on the basis of the features modeled in the simulations support the validity of the model and highlight the potential of this approach. Consequently, the calculations here are effective for the design and implementation of materials that display multiple modes of deformation needed to achieve advances in soft robotics, haptic devices, and “smart,” dynamically adaptive surfaces.

## METHODS

The elastomeric posts are modeled using a finite element mesh, and the equations for the free energy are solved numerically to advance the coordinates of the mesh nodes in time. We use initially cubic finite elements where polynomial shape functions can be used to interpolate any physical quantity at the nodes to find a value in the interior of the element. We developed this scheme for our previous studies (*19*) and have now developed additional code to model the ray tracing. In contrast to tetrahedral finite elements, where the strain tensor is uniquely determined by the displacement of the four vertices, our approach gives a strain tensor that varies within each voxel. Consequently, this quantity varies continuously within the overall mesh, rather than jumping discontinuously between elements.

The free energy for each element is approximated using a Gaussian integration scheme, evaluating the functional in Eqs. 1 and 2 at eight points inside the element. These positions are used as the query points in the ray-tracing algorithm for Eq. 4, which defines the change in the order parameter necessary to compute the free energy in Eq. 2. This energy is then differentiated with respect to the mesh node coordinates to compute a force. Accelerations and positions of each node in the finite element mesh are updated in a leapfrog integration scheme, along with the velocities, at each time step. This process is repeated until the forces from the elastic free energy balance the forces from the light-induced change in the nematic order.

To confirm the computational results, we fabricated surface-anchored square LCE microposts of dimensions 150 μm × 30 μm × 30 μm with the magnetic field–defined director orientation at 45° from vertical in the *xz* plane, thus matching the simulation studies. The system consists of 4′′-acryloyloxybutyl 2,5-di(4′-butyloxybenzoyloxy) benzoate as the side-on monomer and 4,4′-bis(6-acryloyloxynonyloxy) azobenzene (7.5 weight %) as a cross-linker (*19*).

The light-responsive deformation of LCE microposts containing azobenzene cross-linkers was characterized by using confocal fluorescence microscopy (Zeiss LSM 700). Rhodamine B acrylate dye was coated on top of the LCE microstructures for fluorescence microscopy imaging. During the characterization, a UV light-emitting diode (Thorlabs, M365F1) was placed 1 cm from the sample and 0° or 180° to the LCE micropost with respect to the *y* axis (15.8 mW/cm^2^), and the temperature of the sample was kept at 60°C to retain the elastomer above the glass transition temperature in the elastic state.

## Supplementary Material

aay5349_SM.pdf

aay5349_Movie_S1.mp4

## SUPPLEMENTARY MATERIALS

Supplementary material for this article is available at http://advances.sciencemag.org/cgi/content/full/6/13/eaay5349/DC1 Fig. S1. Selection of mesh size. Fig. S2. Time evolution of bending. Fig. S3. Twisting versus height for a post with a tilted nematic director in the *xz* plane. Fig. S4. Diagram of effect of shadowing on twist. Movie S1. Top view of light-responsive deformation of LCE micropost.
