# Supplementary material for: Twist again: Dynamically and reversibly controllable chirality in liquid crystalline elastomer microposts
Source: Sci Adv. 2020 Mar 27;6(13):eaay5349. doi: 10.1126/sciadv.aay5349 (PMC7101207; doi:10.1126/sciadv.aay5349)
Supplement: aay5349_SM.pdf [file aay5349_SM.pdf]

Science Advances
AAAS

advances.sciencemag.org/cgi/content/full/6/13/eaay5349/DC1

Supplementary Materials for

Twist again: Dynamically and reversibly controllable chirality in liquid crystalline elastomer microposts

James T. Waters, Shucong Li, Yuxing Yao, Michael M. Lerch, Michael Aizenberg, Joanna Aizenberg, Anna C. Balazs*

*Corresponding author. Email: balazs@pitt.edu

Published 27 March 2020, *Sci. Adv.* **6**, eaay5349 (2020)
DOI: 10.1126/sciadv.aay5349

The PDF file includes:

Fig. S1. Selection of mesh size.
Fig. S2. Time evolution of bending.
Fig. S3. Twisting versus height for a post with a tilted nematic director in the *xz* plane.
Fig. S4. Diagram of effect of shadowing on twist.
Legend for movie S1

Other Supplementary Material for this manuscript includes the following:

(available at advances.sciencemag.org/cgi/content/full/6/13/eaay5349/DC1)

Movie S1 (.mp4 format). Top view of light-responsive deformation of LCE micropost.

Supporting Information

Calculation of attenuation length

The attenuation length inside the illuminated material can be predicted theoretically using the Beer-Lambert law. The intensity I is assumed to fall off exponentially with respect to the distance l inside the material, giving the expression

$$\log(\frac{I_0}{I}) = \varepsilon c l \tag{1}$$

Here I_0 is the initial intensity, c is the concentration of the absorbing material, and ε is a molar attenuation coefficient. The product $(\varepsilon c)^{-1}$ gives a length scale (λ) over which the intensity falls off within the sample. The fabricated LCE's used to calibrate the simulations parameters have an azobenzene concentration of 0.14 mol/L, and the value of ε is taken to be 2.0×10^4 L / mol cm, giving a predicted λ value of 3.5 µm. This is comparable in magnitude to the value of 6 µm obtained from matching simulation results against experimentally measured values. It is worth noting that the posts used in these experiments were circular in cross section.

Choosing Mesh Size

The mesh resolution is chosen such that each finite element represents a region of the elastomer that is initially cubic in shape, and 2.5 µm on a side. This density of mesh nodes and elements is chosen by taking one value of the incident light angle and penetration depth (as shown in Fig. 1C) and repeating the simulation at a range of mesh resolutions. The underlying assumption is that as the mesh is refined, the results will converge on a single value. We see in fig. S1 that there is little increase in accuracy to be gained for a mesh size finer than 2.5 µm.

Time evolution of bending

Figure S2 displays the time evolution of the bending angle for the posts with a vertically oriented nematic director (Fig. 2). Generally, taller posts take a longer time to relax mechanically, owing to their larger rotational moment with respect to the base. Relaxation is expected to take on the order of minutes experimentally, however the correlation between simulation time steps and physical time is imprecise due to simplifications in the dynamics of the model.

Twisting Profile versus Height

For a nematic director oriented in the *xz*-plane, at 45° from the vertical axis, the nematic vector is expressed as

$$\hat{\mathbf{n}} = \frac{1}{\sqrt{2}}\hat{\mathbf{e}}_x + \frac{1}{\sqrt{2}}\hat{\mathbf{e}}_z \tag{2}$$

For this geometry, we find that the twist per unit height is largest when the azimuthal angle of the incident light is 90° from the plane of the director, along the *y*-axis. This orientation of the incident light, however, does not produce the greatest overall twist, as seen in Fig. 4B, and

shown in detail over the height of the post in fig. S3. As the post in this case twists at its base, the illuminated face moves into shadow and thus, does not contribute to further deformation (see fig. S4). On the other hand, if the incident light is at an azimuthal angle of 125° from the *xz*-plane, then the twisting maintains the illuminated face in the light and hence, this structure can undergo further twisting.

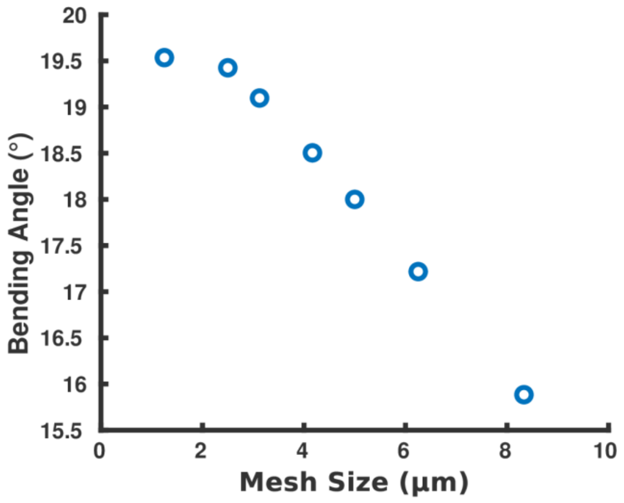

Fig. S1. Selection of mesh size. As the mesh resolution grows finer, the bending angle approaches a constant value. We select the coarsest mesh size (fewest nodes) that gives accurate results, to minimize computational time.

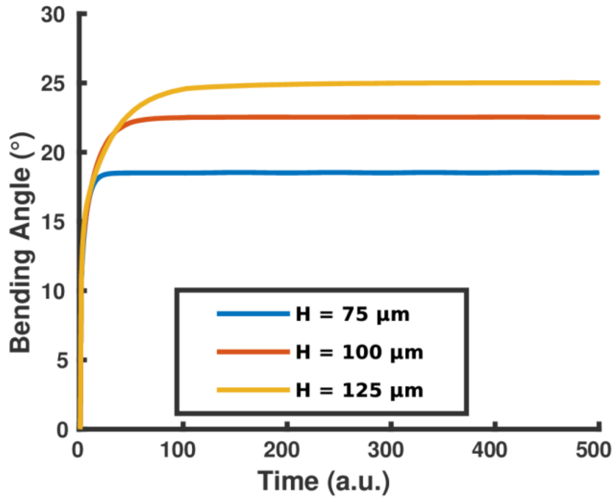

Fig. S2. Time evolution of bending. The data displayed above correspond to the bending angle versus aspect ratio in Fig. 2 of the text. Taller posts are expected to take a longer time to achieve equilibrium.

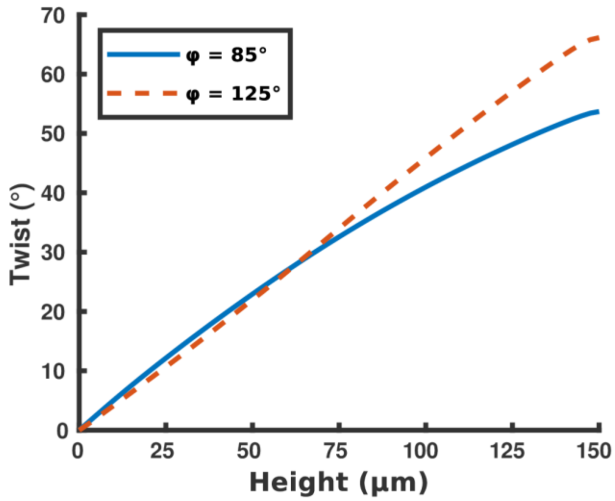

Fig. S3. Twisting versus height for a post with a tilted nematic director in the *xz* plane. For an incident azimuthal angle of 85° (solid blue curve), there is a large twisting rate near the base, but the rate of twisting decreases further up as the twisting exposes another face of the post to the light. When the light source is rotated around the post in the direction of the twist to 125° (dashed orange curve) there is less twisting near the base, but ultimately greater twisting at the top of the post, as the twisting at the base exposes a favorable face of the post to the light.

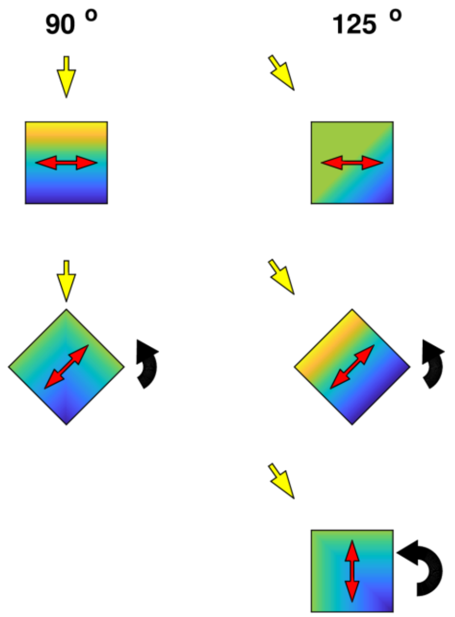

Fig. S4. Diagram of effect of shadowing on twist. For a nematic director tilted in the *xz*-plane, light incident at 90° (along the *y*-axis) will produce the greatest twist per height. However, as the post twists, the nematic director is no longer at a favorable orientation to produce further twisting, as seen at left. When the light source is rotated past 90° in the direction of the twist, as shown on the right, small twisting brings the post into an orientation that produces additional twisting.

Movie S1. Top view of light-responsive deformation of LCE micropost. The UV-responsive microposts showed left-handed twisting and right-handed twisting with incident light from two opposite directions.
